# Facility-level determinants of quality routine intrapartum care in Afghanistan

**DOI:** 10.1186/s12884-021-03916-0

**Published:** 2021-06-23

**Authors:** Megan M. Lydon, Farzana Maruf, Hannah Tappis

**Affiliations:** 1 FHI 360, Durham, NC USA; 2 Athena Institute, Faculty of Science, Vrije Universiteit Amsterdam, Amsterdam, The Netherlands; 3 Global Financing Facility, World Bank Group, Kabul, Afghanistan; 4 Jhpiego, 1615 Thames St, Baltimore, MD USA

**Keywords:** Afghanistan, Maternal health, Childbirth, Quality of care, Respectful maternity care, Health systems, Gender

## Abstract

**Background:**

Although there have been notable improvements in availability and utilization of maternal health care in Afghanistan over the last few decades, risk of maternal mortality remains very high. Previous studies have highlighted gaps in quality of emergency obstetric and newborn care practices, however, little is known about the quality of routine intrapartum care at health facilities in Afghanistan.

**Methods:**

We analyzed a subset of data from the *2016 Afghanistan Maternal and Newborn Health Quality of Care Assessment* that comprised of observations of labor, delivery and immediate post-partum care, as well as health facility assessments and provider interviews across all accessible public health facilities with an average of five or more births per day in the preceding year (N = 77). Using the Quality of the Process of Intrapartum and Immediate Postpartum Care index, we calculated a quality of care score for each observation. We conducted descriptive and bivariate analyses and built a multivariate linear regression model to identify facility-level factors associated with quality of care scores.

**Results:**

Across 665 childbirth observations, low quality of care was observed such that no health facility type received an average quality score over 56%. The multivariate regression model indicated that availability of routine labor and delivery supplies, training in respectful maternity care, perceived gender equality for training opportunities, recent supervision, and observation during supervision have positive, statistically significant associations with quality of care.

**Conclusions:**

Quality of routine intrapartum care at health facilities in Afghanistan is concerningly low. Our analysis suggests that multi-faceted interventions are needed to address direct and indirect contributors to quality of care including clinical care practices, attention to client experiences during labor and childbirth, and attention to staff welfare and opportunities, including gender equality within the health workforce.

**Supplementary Information:**

The online version contains supplementary material available at 10.1186/s12884-021-03916-0.

## Background

There has been an important shift in maternal health efforts in recent decades, expanding in focus from access to facility-based care to also encompass quality of care. In places where access remains a barrier, this shift has been slower to occur. Additionally, when financial resources are limited, it may seem most efficient to focus on access, however, evidence has shown that improved access to obstetric care has not yielded commensurate decreases in maternal mortality across many low-income countries [1, 2]. Conflict-affected areas are particularly prone to challenges of health care access and limited financial resources. Even in these circumstances, attention to quality of care can make an important contribution to population health. In line with this, the World Health Organization (WHO) calls for a focus on quality of care in fragile and conflict-affected areas [3]. As such, it is critical for maternal health programs in conflict-affected areas to assess quality gaps and develop strategic plans to address them.

After the fall of the Taliban in 2001, Afghanistan’s Ministry of Public Health (MOPH) faced the challenge of rebuilding a devastated health system. Available, accessible, quality, and equitable health services were severely lacking [4]. At this time, Afghanistan logged the highest maternal mortality ratio on record [5]. Under Taliban rule, women were denied education which consequently prevented the training of female midwives and obstetricians [5, 6]. In a society where it is considered inappropriate to seek obstetric care from a male provider, this gap in female providers left many women without culturally appropriate options for skilled birth attendance [5, 6].

Over the last two decades, the MOPH has worked to strengthen the country’s health system and made maternal and newborn health a priority. Afghanistan has made notable strides in training female midwives [7], improving access to obstetric care [8], and reducing maternal mortality [9]. Maternal mortality ratio estimates have indeed declined from 1,450 in 2000 to 638 in 2017 but remain unacceptably high [10]. There continue to be shortages across the health workforce, and particularly among female providers [11]. Ongoing conflict and insecurity exacerbate strains on the country’s donor-dependent public health system [12], and discrimination against women and girls is recognized as a persistent problem that affects both health service provision and care seeking [11].

Given socio-cultural norms, all maternity providers in Afghanistan are female, however, they remain a minority amongst the broader health workforce dominated by men [11]. Ethnographic studies in a tertiary hospital providing women’s health services in Kabul capture myriad challenges facing maternity care providers, including complex multi-faceted power dynamics [13, 14]. Conservative gender norms and discrimination are pervasive such that female health workers may face stigma, violence, and security risks both inside and outside of the workplace [11].

While the health system and its providers experience many challenges, service utilization is rapidly increasing. Skilled birth attendance has increased from 34% in 2010 to 51% in 2015, almost exclusively through institutional deliveries [15, 16], yet little is known about the quality of intrapartum care available. A recent qualitative study examining women’s experiences during childbirth at primary health care facilities in eastern Afghanistan suggests quality of care is highly variable [17]. We are not aware of any studies to date quantifying the quality of clinical care practices during routine labor and delivery in Afghanistan.

Several studies have noted quality gaps in emergency obstetric and neonatal care across Afghanistan [18–22], however, less attention has been focused on routine intrapartum care. Strengthening routine obstetric care is essential as it represents the care afforded to the majority of laboring women, and it ensures performance of clinical practices that can prevent complications and ensure timely identification of emergency cases. Quality can be assessed at many levels however recent research suggests that facility-level factors, not characteristics of individual providers, are the primary drivers of intrapartum quality [23]. The culture and environment at a health facility is critical in determining the level of care provided there and individual provider behavior tends to conform to broader facility norms [23].

Accordingly, this study seeks to understand the facility-level drivers of quality of care during routine labor and childbirth at public health facilities in Afghanistan, as measured by the appropriate performance of clinical intrapartum tasks. This focus was selected in order to provide actionable insights to decision-makers about how best to modify health system inputs to strengthen quality of care, leading to improvements in maternal and newborn outcomes.

## Methods

We analyzed a subset of data from the *2016 Afghanistan Maternal and Newborn Health Quality of Care Assessment* to examine the quality of routine intrapartum care and to identify facility-level determinants of quality care at high-volume public-sector health facilities across Afghanistan. The original study was a cross-sectional assessment examining readiness to provide routine care and address major obstetric and newborn complications at public health facilities across all 34 provinces of Afghanistan [24]. The subset of data used in this analysis includes facility inventory and document review checklists, interviews with maternity care providers and observations of normal vaginal births at all 77 accessible public hospitals in Afghanistan with an average of five or more births per day reported in the national health management information system in 2015. This threshold was selected based on WHO Health Facility Assessment Guidelines for estimation of quality of care indicators with ± 10% precision, and past experience of quality of care assessments, as a practical threshold to ensure that data collectors would be able to observe multiple births over a 2–3-day facility visit [25]. These 77 facilities represent a census of accessible high volume public health facilities in Afghanistan (40 district hospitals and comprehensive health centers, 27 provincial hospitals, 5 regional hospitals and 5 specialized hospitals). Two additional district hospitals reported an average of five or more births per day in 2015 but were not accessible due to insecurity at the time of data collection. In the year preceding the assessment, the 77 public facilities visited accounted for 63% (440,097) of all births (669,520) reported in the health management information system.

### Data collection

Data collection was completed during 2–3-day visits to each facility between 14 May and 3 August 2016. Teams of three data collectors, all female Afghan doctors or midwives trained on study tools and research methods, aimed to observe up to five vaginal births and five postnatal examinations at each district hospital and up to 10 vaginal births (five during a day shift and five during a night shift) and five postnatal examinations at each provincial, regional and specialized hospital. The number of provider interviews conducted at each facility (1–8 interviews) was dependent on the number of maternity staff assigned to that facility type and available at the time of data collection.

Data collection tool content was based on WHO guidelines and adapted from tools used in conducting quality of care assessments in other countries [26]; as well as tools used in Demographic and Health Survey Service Provision Assessments (SPA) [27], and Emergency Obstetric and Newborn Care (EmONC) assessments supported by the Averting Maternal Death and Disability program [28]. All tools were developed in English, translated into Dari and Pashto, and pre-tested during data collector training at a public hospital in Kabul [24]. Provider interviews covered previous training received, services provided, supervision received, and current work conditions. Facility assessments included general readiness and labor and delivery service readiness, documenting services available, performance of EmONC signal functions, and availability of supplies, equipment, and medications. Labor and delivery observations include sections on initial client assessment, first stage of labor, second and third stage of labor, as well as immediate newborn and postpartum care.

Data collection was conducted using CommCare software loaded on Android tablets, with paper tools used as backup in sites where use of tablets was considered a security risk or unacceptable to care providers or women. When paper tools were used, data from completed checklists were entered into the software when data collectors were in a safe location with internet access.

### Data analysis

Quality of care was quantified using the validated Quality of the Process of Intrapartum and Immediate Postpartum Care index [29], henceforth referred to as the quality of care (QoC) index. As HIV prevalence is relatively low in Afghanistan, we employed the 19-item index like Tripathi and colleagues did with data in Kenya for validation of the index [29]. The QoC index includes key actions from initial client assessment through labor and the immediate postpartum period. Women in our sample arrived at the facility at various stages of labor and were also referred to the operating room as emergencies presented, interrupting observation. As such, the denominator of the QoC index was adjusted to reflect only those items that could have been achieved given the stages of labor and childbirth observed. There was less than 10% missing data for each of the variables used in the creation of the QoC index across observations; if a response to an index item was missing for a phase that was observed, the item was considered unobserved, resulting in no points awarded for that action. To compare the birth observations with varied numbers of stages of labor and childbirth observed, we converted the score to a fraction of the total possible index items for each case. A final quality score was assigned to each birth observation ranging from 0–1 and is sometimes presented in the findings below as a percent for ease of interpretation.

Facility characteristics were linked to each birth observation that took place at that facility. As noted above, the number of maternity care provider interviews varied based on the size of the facility. As such, responses were collapsed to represent a proportion of providers at each facility who provided a positive response to any yes/no questions and thereby reflect the general experience clients may have with providers at a given health facility. For questions that were based on a scale, the average response of providers at each facility was established. Providers were also asked if they had ever received supervision and if so, whether the last visit occurred within the last three months or not. Each of these options was assigned a score (no supervision = 0, supervision more than 3 months ago = 1, supervision within last 3 months = 2) and then averaged across the providers at each facility. We developed a 7-point score reflecting availability of supplies and equipment needed for routine labor and delivery care, such that a facility was awarded a point for each item recorded as available including partograph, uterotonics, syringes and needles, sterile scissors or blade for cord cutting, clean towels to wrap newborn, gloves, and mask and bag for newborn resuscitation. We used a score for the number of Basic Emergency Obstetric and Newborn Care (BEmONC) signal functions (0–7) performed within the last three months to indicate capacity to address emergencies if/when they occur.

**Fig. 1:**
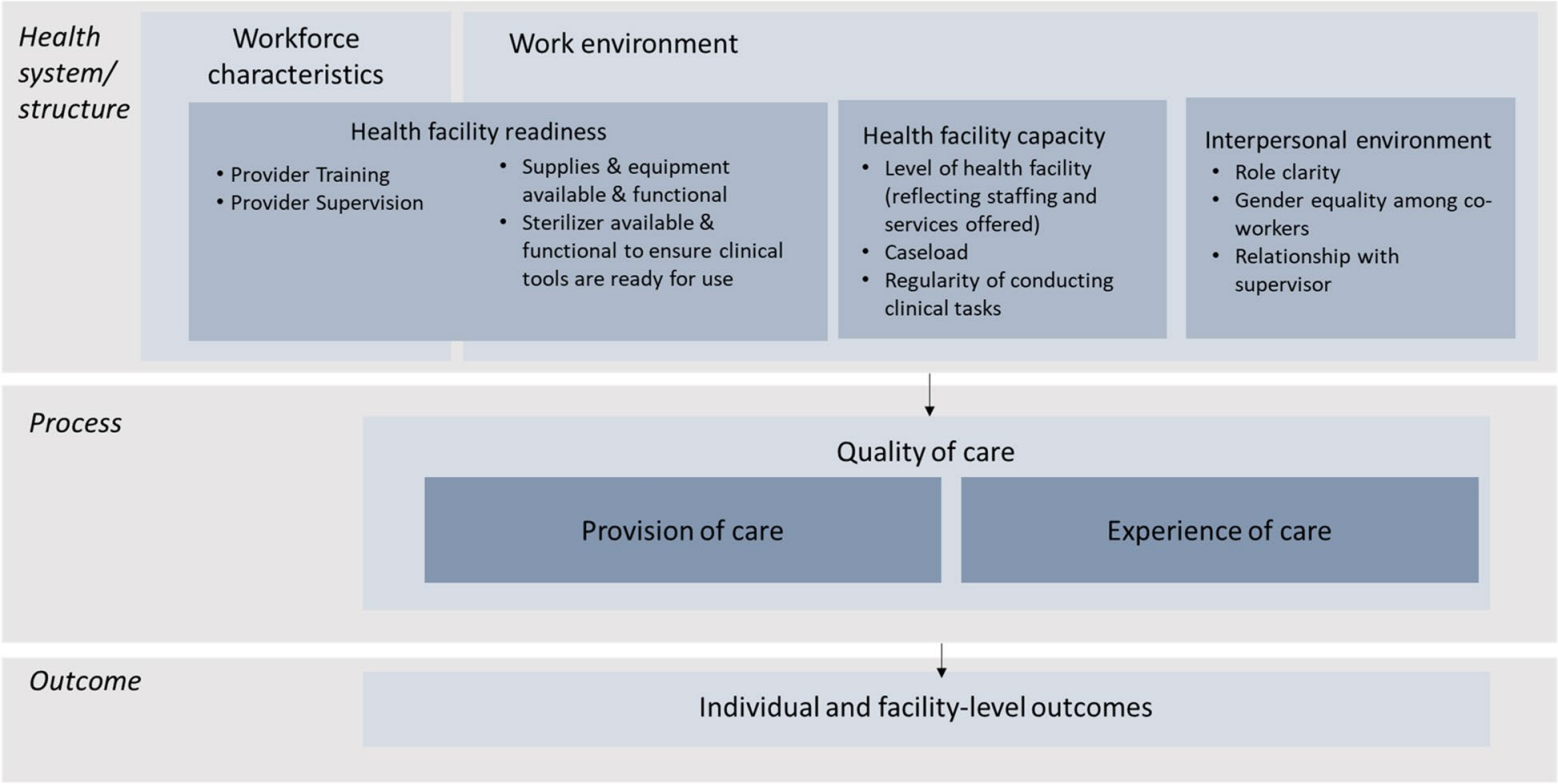
Quality of care framework

Analysis was conducted using Stata version 15 [30]. Descriptive analyses were completed to summarize index items and QoC scores across health facility type. Next, to identify relationships between facility or provider characteristics and quality of care scores, bivariate analyses were conducted and then a multivariate linear regression model was built. Model building was guided by a conceptual framework based on the WHO Quality of Care Framework for Maternal and Newborn Health and the Donabedian model for assessment of quality of care (see Fig. 1 below) [31, 32]. This analysis focuses on “provision of care” as the outcome of interest and examines several health system domains as predictors of this outcome. Domains were included based on previous research and available data. Variables were considered for inclusion in the model if they were deemed theoretically relevant or had a *p-*value < 0.1 in bivariate analysis. Discrete models were built for each conceptual area such as workforce characteristics, interpersonal work environment, and health facility readiness. Factors in each of these models that continued to be significant predictors of quality of care scores were then entered into a backwards stepwise regression model, locking contextually critical variables such as health facility type. Models were assessed and adjusted based on the variance inflation factor, R-squared, and AIC.

## Results

A total of 564 providers were interviewed and 665 women were observed during labor and delivery. Observations included took place at 77 public health facilities, with 9% (61) at the 5 specialized hospitals, 9% (57) at the 5 regional hospitals, 41% (271) at the 27 provincial hospitals and 41% (276) at the 40 district hospitals or comprehensive health centers (CHCs) with five or more births per day. Average caseload across all health facilities sampled was 504 births/month in the year preceding the assessment (see Table 1 for caseload across facility type). Providers interviewed included both midwives and doctors, all female, with a median of 5 years of experience since graduation (range: 0–45 years).

**Table 1:** Health facility labor and delivery caseload statistics

Health facility type	n	Average caseload (births/month)	Caseload range (births/month)
Specialized hospitals	5	1346	988–1659
Regional hospitals	5	1574	1167–2157
Provincial hospitals^a^	26	448	76–1186
District hospitals and CHCs (5 + births per day)	40	228	142–1233

^a^Caseload data missing for one provincial hospital

Quality of care index items were assessed for all 665 labor and delivery observations across the 77 public health facilities (see Table 2 below). Sample sizes vary at different stages of labor as women were admitted to the facility or referred to emergency care at different times. A couple of the QoC index items were widely implemented such that a uterotonic drug was prepared for use in active management of the third stage (AMTSL) of labor for 90% of women, and newborns were immediately dried with a towel in 87% of cases. However, the vast majority of QoC actions were infrequently implemented. Implementation of initial client assessment and examination was low such that providers rarely asked women if they experienced headaches or blurred vision (13%), vaginal bleeding (30%), or took their pulse (31%). Only 33% of women ever received an explanation from a provider of what would happen to them in labor and only 29% of women had their vital signs assessed 15 min after birth. There was variation in the level of implementation of QoC index items across health facility type, with regional hospitals generally underperforming.

**Table 2:** Quality of care index items by health facility type (*n* = 665)

Quality of care index	Secondary and Tertiary Hospitals			Primary Care Facilities	Total average
	Specialized Hospitals	Regional Hospitals	Provincial Hospitals	District Hospitals and CHCs	
*Initial client assessment and examination*	*n* = *27*	*n* = *40*	*n* = *195*	*n* = *189*	***n* = *451***
1. Asks whether woman has experienced headaches or blurred vision	15% (4)	2% (1)	12% (23)	16% (31)	**13% (59)**
2. Asks whether woman has experienced vaginal bleeding	33% (9)	7% (3)	29% (56)	36% (68)	**30% (136)**
3. Takes blood pressure	78% (21)	30% (12)	61% (120)	65% (123)	**61% (276)**
4. Takes pulse	67% (18)	27% (11)	27% (53)	31% (58)	**31% (140)**
5. Washes his/her hand before any examination	56% (15)	42% (17)	37% (72)	37% (70)	**39% (174)**
6. Wears high-level disinfected or sterile gloves for vaginal examination	74% (20)	47% (19)	66% (128)	73% (138)	**67% (305)**
*First stage of labor*	*n* = *43*	*n* = *39*	*n* = *213*	*n* = *220*	***n* = *515***
7. At least once, explains what will happen in labor to the woman and/or her support person	35% (15)	20% (8)	33% (70)	35% (77)	**33% (170)**
8. Prepares uterotonic drug to use for AMTSL	98% (42)	79% (31)	88% (187)	93% (205)	**90% (465)**
9. Uses partograph (during labor)	74% (32)	36% (14)	42% (89)	60% (133)	**52% (268)**
10. Self-inflating ventilation bag (500 mL) and face masks (size 0 and size 1) are laid out and ready for use for neonatal resuscitation	21% (9)	13% (5)	27% (58)	30% (67)	**27% (139)**
*Third stage of labor*	*n* = *61*	*n* = *57*	*n* = *271*	*n* = *273*	***n* = *662***
11. Correctly administers uterotonic (timing, dose, route)^a^	52% (32)	28% (16)	40% (108)	37% (100)	**39% (256)**
12. Assesses completeness of placenta and membranes	56% (34)	32% (18)	57% (154)	49% (135)	**51% (341)**
13. Assesses for perineal and vaginal lacerations	80% (49)	56% (32)	75% (203)	68% (185)	**71% (469)**
*Immediate newborn and postpartum care*	*n* = *61*	*n* = *56*	*n* = *263*	*n* = *272*	***n* = *652***
14. Immediately dries baby with towel	92% (56)	82% (46)	87% (230)	87% (238)	**87% (570)**
15. Places newborn on mother’s abdomen skin-to-skin	64% (39)	36% (20)	47% (124)	48% (131)	**48% (314)**
16. Ties or clamps cord when pulsations stop, or by 2–3 min after birth (not immediately after birth)	88% (54)	66% (37)	70% (183)	71% (194)	**72% (468)**
17. Takes mother's vital signs 15 min after birth	25% (15)	11% (6)	30% (78)	34% (92)	**29% (191)**
18. Palpates uterus 15 min after birth	44% (27)	20% (11)	48% (126)	50% (135)	**46% (299)**
19. Assists mother to initiate breastfeeding within one hour	28% (17)	34% (19)	34% (89)	44% (119)	**37% (244)**

^a^ Correct administration of uterotonic included 10 IU of oxytocin intramuscularly within 1 min of delivery, 600 μg of misoprostol orally within 1 min, or 200 μg of ergometrine intramuscularly within 1 min. Results here are lower than previously published findings from this assessment as Ansari and colleagues examined a subset of these criteria [19]

Quality of care scores were calculated for each labor and delivery observation and averaged across health facility type and group (see Table 3). There was a significant difference across health facility type (*p *< 0.0001) but no significant difference between primary and secondary/tertiary facilities (*p *= 0.115). Of note, no health facility type had an average quality of care score above 56%.

**Table 3:** Average quality of care score, by health facility type and group (*n* = 665)

	**Number of observations**	**Mean quality of care score (scale 0–1)**	**Standard deviation**
**Secondary and tertiary hospitals**	**389**	**0.50**	**0.20**
Specialized Hospitals	61	0.56	0.16
Regional Hospitals	57	0.37	0.20
Provincial Hospitals	271	0.51	0.20
**District hospital or CHC (5 + births/day)**	**276**	**0.52**	**0.24**
**All facilities**	**665**	**0.51**	**0.22**

Associations between provider experience at health facilities and quality of care scores were examined (see Table 4). In terms of training, the proportion of providers who reported receiving related clinical training in the last three years, including any pre- or in-service labor and delivery training, BEmONC training, and training in newborn resuscitation, was not significantly associated with quality of care scores at the same facility. Only training in essential newborn care was associated, such that as the proportion of providers reporting being trained in essential newborn care increases at a facility, so too does the quality of care score (effect size 7.9% [95% CI = 1.1%-14.7%], *p *= 0.023). Proportion of providers trained in quality improvement approaches was not significantly associated with quality of care scores however training on respectful maternity care (RMC) was associated. As the proportion of providers reporting being trained in RMC at a health facility increases, so too do the quality of care scores at that facility (effect size 9.6% [95% CI = 1.0–18.1%], *p *= 0.028). Similarly, quality of care scores significantly increased with an increasing proportion of providers reporting having received training in gender and human rights (effect size 12.8% [95% CI = 2.9–22.7%], *p *= 0.012).

**Table 4:** Bivariate associations of maternity provider experience with quality of care scores

	β	SE	95% CI	*p-*value
**Training in last 3 years**				
Any pre- or in-service training on labor & delivery	-0.0286	0.0343	(-0.0959—0.0387)	0.4039
BEmONC	-0.0376	0.0433	(-0.1227—0.0474)	0.3852
Essential newborn care	0.0791	0.0347	(0.0109—0.1473)	0.0231
Newborn resuscitation	0.0400	0.0273	(-0.0136—0.0935)	0.1432
Maternal death or near miss review/audit	-0.0353	0.0540	(-0.1413—0.0706)	0.5129
Quality improvement approaches	0.0448	0.0558	(-0.0648—0.1545)	0.4225
HMIS data quality and use	0.1110	0.0584	(-0.0036—0.2256)	0.0576
Respectful maternity care	0.0957	0.0435	(0.0102—0.1812)	0.0283
Gender and human rights	0.1278	0.0505	(0.0286—0.2271)	0.0116
**Supervision**				
Recent supervision of providers^a^	0.0761	0.0249	(0.0273—0.1249)	0.0023
Level of respect providers feel from supervisors	0.0363	0.0185	(0.0000—0.0727)	0.0503
During last supervision:^b^				
Supervisor observed provider’s work	0.2222	0.0406	(0.1425—0.3019)	0.0000
Supervisor gave verbal feedback about work	-0.0661	0.0309	(-0.1269—-0.0053)	0.0331
Supervisor discussed problems provider encountered	-0.0687	0.0316	(-0.1306—-0.0067)	0.0299

^a^Categorical variable (never supervised; supervised in last 3 months; supervised more than 3 months ago) averaged across providers interviewed at each facility

^b^If provider did not report receiving supervision, the activity was categorized as having not occurred

Supervision was another factor explored. There was a significant positive association between the proportion of providers receiving supervision and quality of care scores at health facilities such that facilities with more providers reporting having any or recent supervision also scored higher on the QoC index (effect size 7.6% [95% CI = 2.7–12.5%], *p *= 0.002). Facilities with a higher proportion of providers who felt more respected by their supervisors (scale of 1–5, with 5 being most respected, averaged across providers at each facility), tended to have higher quality of care scores (effect size 3.6% [95% CI = 0.0–7.3%], *p *= 0.050). Providers who had received supervision were asked about the content of their most recent supervision visit. At facilities where more providers reported that their supervisor had observed their work, quality of care scores were significantly higher (effect size 22.2% [95% CI = 14.2–30.2%], *p *< 0.001), however, where more providers reported receiving verbal feedback or discussing problems with supervisors, quality of care scores were significantly lower (see Table 4).

Associations between health facility work environment characteristics and quality of care were also assessed (see Table 5). Availability of routine labor and delivery supplies (*p *< 0.0001) and the ability to sterilize necessary equipment (*p *< 0.0001) were both significantly positively related to quality of care. For each additional element of supplies available, quality of care scores at the same health facility increased by 4.1% (2.9–5.4%). Facilities that had any type of functioning sterilizer available experienced 13.2% (7.5–18.9%) higher quality of care scores than those without. There was a significant negative relationship between facility management of obstetric and newborn complications, as indicated through performance of BEmONC signal functions, and quality of care scores for routine delivery at those facilities (effect size -3.3% [95% CI = -0.7—6.0%], *p *= 0.013). Of interest is the link between various measures of gender equality among providers and the quality of care extended to clients. Providers (all female, per Afghan custom) were asked if they believed they had equal treatment and opportunities as colleagues of the opposite sex across several domains. Facilities where a greater proportion of maternity care providers believed they had gender equality in terms of training opportunities (effect size 12.9% [95% CI = 7.0–18.7%], *p *< 0.001), time off (effect size 12.1% [95% CI = 6.3–17.9%], *p *< 0.001), and work schedule (effect size 10.6% [95% CI = 4.3–17.0%], *p *= 0.001), had significantly higher quality of care scores.

**Table 5:** Bivariate associations between work environment characteristics and quality of care scores

	β	SE	95% CI	*p-*value
No. BEmONC signal functions conducted	-0.0334	0.0135	(-0.0598—-0.0069)	0.0134
No. routine labor & delivery supplies available	0.0415	0.0065	(0.0286–0.0543)	0.0000
Any functioning sterilizer	0.1320	0.0292	(0.0747–0.1892)	0.0000
Daily caseload of deliveries	-0.0009	0.0005	(-0.0020—0.0001)	0.0650
Has a written job description for position	0.1909	0.0722	(0.0492—0.3327)	0.0084
*Believes has equal treatment and opportunities as colleagues of the opposite sex in terms of:*				
Training opportunities	0.1288	0.0297	(0.0704—0.1871)	0.0000
Time off	0.1208	0.0296	(0.0626—0.1790)	0.0001
Work schedule	0.1061	0.0323	(0.0427—0.1696)	0.0011
Workload	0.0392	0.0298	(-0.0192—0.0976)	0.1880

The multivariate linear regression model (Table 6) significantly predicts about 24% of the variability in quality of care scores (F < 0.001, R^2^ = 0.239). Holding all other variables constant, health facility type is significantly associated with quality of care (*p *< 0.001) such that provincial and regional hospitals perform worse than district hospitals and CHCs while specialized hospitals perform better. A broad spectrum of variables representing provider and work environment characteristics also significantly predict quality of care scores. In particular, having a greater proportion of providers trained in respectful maternity care had a large significant positive effect on quality of care (18.6% [7.5–29.8%]). Feeling there is greater gender equality among providers in terms of opportunities for training, having more necessary supplies available, having more recent supervision, increased proportion of supervisors observing providers’ work, and having a written job description all had significant positive effects on quality of care scores. As with the bivariate associations above, increased proportion of supervisors providing verbal feedback continues to have a significant negative effect on quality of care scores.

**Table 6:** Multivariate linear regression model of quality of care (n = 665)

	β	SE	95% CI	*p-*value
Health facility type (*Ref.* District hospital or CHC 5 +)				0.000
Provincial hospital	-0.039	0.017	(-0.071—-0.006)	0.021
Regional hospital	-0.198	0.030	(-0.257—-0.139)	0.000
Specialized hospital	0.078	0.029	(0.020—0.136)	0.008
Any pre- or in-service training on labor and delivery in last 3 years	-0.080	0.045	(-0.169—0.009)	0.079
Respectful maternity care training in last 3 years	0.186	0.057	(0.075—0.298)	0.001
Believes has equal treatment and opportunities as colleagues of the opposite sex in terms of training	0.099	0.029	(0.041—0.157)	0.001
No. BEmONC signal functions	-0.024	0.013	(-0.048—0.001)	0.060
No. routine labor & delivery supplies available	0.038	0.007	(0.025—0.052)	0.000
Recent supervision of providers	0.088	0.025	(0.040—0.136)	0.000
Last time supervised, supervisor gave verbal feedback about work	-0.135	0.029	(-0.193—-0.078)	0.000
Last time supervised, supervisor observed provider’s work	0.231	0.038	(0.156—0.306)	0.000
Has a written job description for position	0.160	0.072	(0.019—0.301)	0.026

## Discussion

This study shows low overall quality of intrapartum care across public health facilities in Afghanistan. These results are concerning such that no facility type reached an average quality of care score above 56%. Frequency of performance of many items was similar to that observed in four African countries where the index was validated [29], and overall scores were slightly lower than those measured in SPA surveys including direct observation of intrapartum care in Kenya and Malawi [33]. However, quality of care was variable across health facility type and stage of labor within each facility type. A closer examination of the quality of care indicators highlighted that many of the tasks least frequently implemented were related to routine client monitoring – assessment of headaches, blurred vision, or vaginal bleeding at intake and maternal vital signs postpartum. Inattention to these clinical monitoring tasks could lead to delayed identification of complications and provision of emergency care.

The detection of low overall quality of care prompted further analysis to identify facility-level determinants to better understand contributing factors and potential areas for public health intervention. The analysis highlighted the spectrum of contributors to quality of care in this context. Indicators across all quality of care domains explored, including provider training, supervision, availability of necessary supplies and equipment, and work environment, remained independent predictors of quality of care. This finding emphasizes a need for a health systems approach to improve intrapartum quality of care – no one domain is solely responsible for quality of care scores here but rather a combination of these interconnected domains.

The role of supervision in the regression model of quality of care presented here warrants further discussion. Three supervision indicators remained significant predictors in the final model and help to highlight distinctions in the implementation of supervision. The results show that having more recent supervision is associated with better quality of care, which is unsurprising, however, an examination of the content of that supervision provides more nuanced insights. While observing providers’ work is beneficial, verbal feedback was associated with lower quality of care. This may indicate that supervisors tend to share feedback with those making errors and generally underperforming. It could also be reflective of different types of supervision practices – supervision with the goal of assessing a provider’s performance, compared to supervision with the goal of coaching or mentoring [34]. The results suggest that a focus on supportive supervision which emphasizes learning rather than evaluation, with adequate frequency, may lead to better quality care outcomes.

One of the most striking findings in this study was the association of respectful maternity care training with higher quality of care. Even when accounting for clinical trainings and a plethora of health facility characteristics, RMC training remained a large significant independent predictor of quality care. One indicator in the QoC index—*at least once, explains what will happen in labor to the woman and/or her support person* – speaks directly to the principles of respectful care. However, the proportion of providers that received RMC training at a facility was not correlated with this QoC index item. This suggests that respectful maternity care training may directly lead to improved provision of clinical quality of care. The World Health Organization has underscored the importance of including respectful maternity care within the scope of quality of care to uphold human rights and client dignity [31]. Research has also highlighted the need to work towards providing positive labor and delivery experiences as it influences client decision-making about facility-based care [35]. Striving for respectful maternity care to reach these goals is valuable, and the findings presented here suggest there may be an additional reason to prioritize RMC. Training providers in RMC may have the unanticipated benefit of also improving clinical care. The mechanism of action is unclear however one potential pathway could be through more focused attention on the routine needs of the client which may lend itself to increased conduct of routine client monitoring. Another possible mechanism is that RMC training helps reduce discrimination and stigma that may otherwise lead to inequitable quality of care. Studies have noted that women of younger age and lower education experience higher levels of disrespect and abuse during labor and delivery [36]. In Afghanistan, qualitative research has demonstrated that respectful maternity care is a gap that must be addressed [13, 17]. If providers are better able to recognize their unconscious bias and actively attempt to engage in more equitable care, clients will benefit from improved clinical care. Further research is needed to replicate these findings and explore the possible mechanisms of action linking RMC interventions to clinical quality of care.

Another notable finding of this study was the association of perceived gender equality among providers with increased provision of quality care. While providers must be afforded opportunities to improve their skills and knowledge, they must also be enabled to do their best work through a conducive work environment. This does not simply entail infrastructure, supplies, equipment, and medications, it also involves supportive interpersonal dynamics and socio-cultural norms. The findings from this study highlight the importance of gender equality as it contributes to the enabling environment and increased provision of quality care. It has been documented that midwives face gender discrimination and violence [37], are perceived as less competent providers than their male counterparts, and have less decision-making power and mobility [38]. These constraints erode midwives’ ability to provide quality care [39]. In a context such as Afghanistan where women are encouraged to seek care from female providers, it is critical that the female health workforce is afforded the respect, opportunities, and work environment to facilitate their care provision, ensuring gender equity in health care.

While this study has demonstrated the clinical quality improvements related to respectful maternity care training and gender equality among providers, there is a need to explore these relationships in other contexts and through more rigorous study designs. Previous research has noted variability in the effectiveness of provider trainings and the importance of dose, frequency, quality of training, pairing trainings with supervision, and ensuring trainings are not stand-alone events but rather, linked to broader interventions [40, 41]. A recent review of RMC interventions highlights that multifaceted interventions can have positive effects on clinical practice and quality of care, emphasizing the need to address health facility culture, relationships with the community, and larger societal factors [42]. As gender constructs and perceptions of maternity are rooted in broader social and cultural norms, and influenced by the socio-political, economic, and environmental context, effective interventions will likely need to encompass an integrated approach. An important next step will be to harness implementation research and cost-effectiveness studies to identify promising interventions that can alleviate gender inequity and disrespectful care, leading to improvements in women’s experience and quality of care.

## Limitations

There are limitations that should be considered when interpreting the findings of this study and their implications for policy and programming. First, we were unable to link provider interview data with client observations due to study design and logistical challenges in assigning unique IDs to providers. As such, the analyses cannot directly link provider characteristics to the quality of care afforded by an individual provider. However, evidence from other settings suggests that the quality of intrapartum care a patient receives is influenced far more by the facility where she receives care than the health worker who provides care [23]. Second, the study collected limited data on supervision characteristics, such as who conducted the last supervision visit, which could help better understand the nuances of the supervision associations noted in this analysis. We recommend that future assessments collect more detailed information about supervision to provide greater insight on this issue. Last, this study only examined potential determinants of routine intrapartum care that were measured in the *2016 National Maternal and Newborn Health Quality of Care Assessment*’s documentation of facility readiness and work environments; the best fitting regression model, presented here, explains 24% of the variability in QoC scores. This indicates that there are many more elements of quality of care we have yet to accurately measure. In particular, this study did not include measures of client experience or patient-provider communication, due to the limitations of data available. Anecdotal evidence suggests that discrimination based on wealth status, ethnicity, and family/clan relations may be common when providing intrapartum care in the Afghan context [43]. Further research accounting for client, provider, and facility level factors could provide greater clarity on drivers of quality intrapartum care. Despite these limitations, this study provides important evidence on gaps in quality of routine intrapartum care in Afghanistan as well as the facility-level drivers.

## Conclusions

The quality of routine intrapartum care at high-volume public health facilities in Afghanistan must be addressed. Effective interventions will be those that take a health systems approach. For too long, global health efforts have operated through vertical interventions, however, maternal health programs will only be successful if they are part of a broader health system framework. In Afghanistan, this means improved coordination and integration across health sectors is needed. Promising interventions will work to enable respectful maternity care and gender equality in the healthcare workplace. While efforts to improve respectful maternity care and gender equality may initially seem like admirable but supplementary features in an already stretched health system, investment in these areas can have a major direct impact on patient quality of care. It is critical to think of health workforce gender equality and respectful maternity care as key contributors to quality of care that warrant prioritization.

## Supplementary Information

**Additional file 1: Supplementary Table 1**. Proportion of providers reporting receiving various training and supervision experiences.

## Data Availability

Quantitative data are available from the MoPH upon request. Requests should be directed to the MoPH’s Evaluation and Health Information Systems Department (ehis.moph@gmail.com).

## Abbreviations

AMTSL: Active management of the third stage of labor
BEmONC: Basic Emergency Obstetric and Newborn Care
CHC: Comprehensive health centers
DH: District hospital
EmONC: Emergency Obstetric and Newborn Care
HMIS: Health management information system
MOPH: Ministry of Public Health (Afghanistan)
QoC: Quality of care
RMC: Respectful maternity care
SPA: Service provision assessment
WHO: World Health Organization
