# Supplementary material for: Facility-level determinants of quality routine intrapartum care in Afghanistan
Source: BMC Pregnancy Childbirth. 2021 Jun 23;21:438. doi: 10.1186/s12884-021-03916-0 (PMC8223289; doi:10.1186/s12884-021-03916-0)
Supplement: Supplementary file 1 — Additional file 1: Supplementary Table 1. Proportion of providers reporting receiving various training and supervision experiences. [file 12884_2021_3916_MOESM1_ESM.docx]

## **Supplementary Table 1:** Proportion of providers reporting receiving various training and supervision experiences (providers=564, health facilities=77)

|  | Total number of providers (%)† | Mean percent of providers at each health facility (SD) |
| --- | --- | --- |
| **Training in last 3 years** |  |  |
| *Any pre- or in-service training on labor & delivery* | *228 (40.4)* | 41.6 (25.9) |
| BEmONC | 131 (57.5) | 22.4 (20.1) |
| Maternal death or near miss review/audit | 68 (29.8) | 11.9 (15.5) |
| Quality improvement approaches | 69 (30.3) | 11.8 (14.3) |
| HMIS data quality and use | 70 (30.7) | 12.5 (14.1) |
| Respectful maternity care | 105 (46.1) | 18.2 (19.3) |
| Gender and human rights | 73 (32.0) | 12.9 (17.2) |
| *Any pre- or in-service training on subjects related to newborn care* | *192 (34.0)* | 34.7 (26.2) |
| Essential newborn care | 155 (80.7) | 27.7 (24.5) |
| Newborn resuscitation | 168 (87.5) | 30.3 (25.4) |
| **Supervision** |  |  |
| *Recency of supervision* | *464 (82.3)* | *1.68 (0.34)* |
| Never supervised (score=0) | 30 (6.5) |  |
| Supervised more than 3 months ago (score=1) | 81 (17.5) |  |
| Supervised within the last 3 months (score=2) | 353 (76.1) |  |
| *During last supervision‡:* |  |  |
| Supervisor observed provider’s work | 355 (81.8) | 81.8 (20.4) |
| Supervisor gave verbal feedback about work | 261 (60.1) | 63.0 (27.1) |
| Supervisor discussed problems provider encountered | 246 (56.7) | 61.8 (27.3) |

†Due to skip patterns, denominators are based on the initial indicators in italics

‡Among those reporting they had ever received supervision (434 providers)
